# Dr. J. Hawkins on the Treatment of Congenital Inguinal Hernia

**Published:** 1844-04-20

**Authors:** 


### DR. J. HAWKINS ON THE TREATMENT OF CONGENITAL INGUINAL HERNIA.—OPERATION.

Mr. J., aged twenty-six, sent for Mr. Whitmore, November 1, 1832, late in the evening, complaining of pain in the bowels, &c.

A tumour, about the size of an egg, situate in the left groin, was discovered—tense and very painful on pressure. He had been sick, and felt slight pain in the bowels: no stool had passed since October 29. After giving some general directions, Mr. W. left him for the night, being in attendance on a dangerous midwifery case. On visiting him the next morning, Mr. W. found him vomiting, with an anxious countenance, and the usual sy ıptoms of strangulated hernia. The warm bath, bleeding from the arm, and moderate use of the taxis, were tried, but the reduction of the hernia was not accomplished; neither testicle had descended into the scrotum.

He gave the following history of his case; He had a rupture as long as he can remember, and about fifteen months since he had a similar attack to the present one, while residing in the country. He sent for a surgeon, who failed in giving him any relief; having dismissed this practitioner, Mr. J. sent for a noted "quack doctor." The case being attended with obstinate constipation, this empiric prescribed and administered a drastic purgative powder, which he called "gold dust," and after each dose he made the patient walk about the room. The rupture was reduced.

About twelve, A. M., Mr. Charles Fowler saw the patient with Mr. Whitmore, and having again endeavoured cautiously to reduce the hernia by the taxis ineffectually, the operation was proposed and consented to by the patient.

Nothing particular took place in its progress till the sac was opened, when an unusual quantity of dark brown fluid escaped; the contents were omentum and intestine; the stricture was divided, and the protruded parts returned into the cavity of the abdomen. This being done, the testicle was found lying in the sac, which at once demonstrated the true nature of the case; it was clearly one of congenital inguinal hernia. The wound was closed, and the man recovered without any untoward symptom manifesting itself.

I have been induced to forward this case to you, because I believe it to be one of unusually rare occurrence; cases of congenital hernia almost invariably descend into the scrotum. I have repeatedly witnessed the performance of operations under such circumstances. A case of congenital scrotal hernia, in which the testicle had not passed the external abdominal ring, was operated on successfully in the Cheltenham Hospital, by Mr. Eves. I consider it would not be justifiable to remove a testicle if healthy during the operation. (I was informed by this man that he was the father of two children, although neither testis had passed into the scrotum.) On the other hand, the presence of the testis must of course prevent the application of a truss and thus the patient is constantly exposed to the dangers of strangulated hernia.—*Prov. Med. Journ. Jan.* 20, 1844.

